# Temporal and spatial relationship of gene expression in the infarcted rat heart

**DOI:** 10.1186/1471-2105-13-S12-A16

**Published:** 2012-07-31

**Authors:** Wenyuan Zhao, Tieqian Zhao, Yuanjian Chen, Yao Sun

**Affiliations:** 1 Division of Cardiovascular Diseases, Department of Medicine, University of Tennessee Health Science Center, Memphis, TN 38163, USA

## Background

Gene interaction plays an important role in regulating the molecular and cellular actions in both the physiological and pathological circumstances. Following myocardial infarction (MI), cardiac repair/remodeling represented as cardiac inflammation, angiogenesis, apoptosis, fibrosis occur in the infarcted and noninfarcted myocardium. Local angiotensin system (RAS) is involved in the cardiac repair.

## Materials and methods

In the current study, thirty-five genes involved in cardiac repair and RAS were detected by Real Time PCR in both the infarcted and non-infarcted myocardium at different time point post-MI. Pearson correlation analysis showed a number of significant correlations between the expressions of these genes. The different phases of cardiac repair/remodeling and regions of the infarcted heart impacted the correlation directions (positive or reverse), correlation efficient value, and corresponding significant levels. Multiple linear regression models fitting also showed that the different phases of cardiac repair/remodeling and different regions of the infarcted heart determined the dependent factors in the models. The interactions among genes were further explored by Ingenuity Pathway Analysis software. We found that several pathological pathways were involved in cardiac repair and remodeling in the different regions of the infarcted heart at different stages postMI.

